# Accuracy of Two Point-of-Care Tests for Rapid Diagnosis of Bovine Tuberculosis at Animal Level using Non-Invasive Specimens

**DOI:** 10.1038/s41598-020-62314-2

**Published:** 2020-03-25

**Authors:** Holden V. Kelley, Sarah M. Waibel, Sabeen Sidiki, Cristina Tomatis-Souverbielle, Julia M. Scordo, W. Garret Hunt, N. Barr, R. Smith, Sayeed N. Silwani, James J. Averill, Susan Baer, Janet Hengesbach, Vedat O. Yildiz, Xueliang Pan, Wondwossen A. Gebreyes, Joan-Miquel Balada-Llasat, Shu-Hua Wang, Jordi B. Torrelles

**Affiliations:** 1 Texas Biomedical Research Institute, San Antonio, TX United States; 2 Department of Microbial infection and Immunity, College of Medicine (COM), The Ohio State University (OSU), Columbus, OH United States; 3 Department of Preventative Medicine, College of Veterinary Medicine, OSU, Columbus, OH United States; 4 Nationwide Children’s Hospital, Division of Pediatric Infectious Diseases, OSU, Columbus, OH United States; 5 Nationwide Children’s Hospital, Division of Infectious Diseases, OSU, Columbus, OH United States; 6 Michigan Department of Agriculture and Rural Development, Lansing, MI United States; 7 Diagnostic Center for Population and Animal Health, Michigan State University, East Lansing, MI United States; 8 Center for Biostatistics, COM, OSU, Columbus, OH United States; 9 OSU Global One Health Initiative, Columbus, OH United States; 10 Department of Pathology, COM, OSU, Columbus, OH United States; 11 Department of Internal Medicine, Division of Infectious Diseases, COM, OSU, Columbus, OH United States

**Keywords:** Infectious-disease diagnostics, Pathogens, Infection

## Abstract

Bovine tuberculosis (BTB) testing in cattle requires a significant investment of time, equipment, and labor. Novel, rapid, cheaper and accurate methods are needed. The Alere Determine TB lipoarabinomannan antigen (LAM-test) is a World Health Organization-endorsed point-of-care urine test designed to detect active TB disease in humans. The Lionex Animal TB Rapid Test (Lionex-test) is a novel animal specific TB diagnostic blood test. An animal level analysis was performed using urine (n = 141) and milk (n = 63) samples from depopulated BTB-suspected cattle to test the accuracy of the LAM-test when compared to results of positive TB detection by any routine BTB tests (BOVIGAM, necropsy, histology, culture, PCR) that are regularly performed by the United States Department of Agriculture (USDA). The agreement between the urine LAM-test and USDA standard tests were poor at varying testing time points. The same milk samples did not elicit statistically significant agreement with the Lionex-test, although positive trends were present. Hence, we cannot recommend the LAM-test as a valid BTB diagnostic test in cattle using either urine or milk. The Lionex-test’s production of positive trends using milk samples suggests larger sample sizes may validate the Lionex-test in accurately diagnosing BTB in cattle using milk samples, potentially providing a quick and reliable field test for BTB.

## Introduction

Bovine tuberculosis (BTB) is a zoonotic disease caused by the bacterium *Mycobacterium bovis*^1,2^. The Cooperative State-Federal TB Eradication Program, including the United States Department of Agriculture (USDA), state animal health agencies, and US livestock producers, have nearly eliminated *M. bovis* infection from cattle in the US^3,4^. Although inspectors test more than one million animals a year for BTB and have taken steps to eradicate this disease, *M. bovis* is still present^5^. Globally, *M. bovis* is typically spread from cattle to cattle but in the US, wildlife (e.g. white tail deer, elk, bison, badgers, etc.) more frequently infect cattle, particularly in Michigan, which is the focus of this study^5,6^. Thus, domestic cattle and wildlife pose a potential threat to human health^5,6^. Indeed, BTB is typically transmitted from cattle to humans primarily through consumption of unpasteurized dairy products or occasionally contaminated meats^2,7,8^. Current diagnostic testing and eradication protocols along with pasteurization have caused the prevalence of BTB to drop significantly in the US as well as in other developed countries^9,10^. In 2016, the World Health Organization (WHO) estimated 147,000 cases of zoonotic TB with 12,500 deaths^11^. Globally, however, the median proportion of *M. bovis* cases of the total TB cases reported in humans ranges from 15.4 to 26.1% in African countries like Ethiopia, Nigeria, and Tanzania^1^.

Diagnosis of BTB is difficult since animals with disease often do not show signs until the infection has reached an advanced stage^8,9^. In some countries, delay in BTB diagnosis may increase transmission rates from animals to humans. BTB eradication programs in developed countries like the US include comprehensive screening of imported and domestic cattle, tracking cattle movement between farms, euthanizing skin test-positive animals (reactors), inspecting meat at slaughter plants, pasteurizing dairy products, and performing positive sample tracebacks. If *M. bovis* is detected in a particular cow from a farm, then all cattle are quarantined and screened for *M. bovis* infection. If infection is confirmed in any of the tested cattle, then whole herd depopulation is performed or individual testing and removal is implemented^8,9^. This method of surveillance and control drives a substantial economic burden. Within the last 10 years, the USDA-Animal and Plant Health Inspection Service (APHIS) program has directed $342 million of its budget on US BTB surveillance and control^4^. This does not include the cost of indemnity payments to farmers, cleaning and disinfection of infected farms, or wildlife surveillance in BTB-infected regions. Likewise, in 2013, the UK government spent £99 million on BTB with 35.6% of cost going towards cattle compensation costs^12^. These labor and cost intensive approaches to reduce the prevalence will not be feasible in developing regions/countries.

Current detection methods rely on moderately sensitive, expensive, and labor intensive intradermal tuberculin tests^13,14^, delayed culturing processes, BOVIGAM (IFN-γ release assay in blood), and/or PCR testing. Present screening methods include the Caudal Fold Tuberculin test (CFT) which is read at 72 h ± 6 h. If the animal responds to the CFT, then this test is followed by the Comparative Cervical Tuberculin test (CCT) also read at 72 h ± 6 h as a confirmatory test. The CCT must be administered by a state or federal veterinarian trained in the application of the test. If required, follow-up CCT testing must be performed within 10 days of the initial CFT in cattle, or the veterinarian must wait 60 days after the injection of the CFT before a follow-up CCT re-testing to avoid desensitization^3^. An animal is considered a reactor if it shows a response to the aforementioned official TB tests and is classified as a reactor by the testing, accredited, veterinarian or designated TB epidemiologist^3^. An animal can also be considered a reactor upon slaughter inspection, necropsy, histology, PCR, and/or culture by the federal or state veterinarian performing or supervising^3^. Compounding this issue is the reluctance of farmers to slaughter suspect BTB cattle for post-mortem testing (necropsy or histology) given the low prevalence of BTB in US cattle and subsequent high rate of false positives. Thus a simple, easy-to-use, cost-effective, and rapid diagnostic test for BTB is in needed to combat this disease.

The Alere Determine TB LAM Ag (LAM-test, Waltham, MA, USA) is a point-of-care (POC) rapid test that uses the *Mycobacterium tuberculosis* complex lipoarabinomannan (LAM) antigen excreted in urine to detect active TB disease in humans^15–18^. Since *M. bovis* is part of the *M. tuberculosis* complex and contains LAM, in this study we determined if LAM could also be detected in the urine and milk of cattle to diagnose BTB.

The Lionex Animal TB Rapid Tuberculosis Test (Lionex-test, Braunschweig, Germany) is another rapid TB test that uses whole blood, serum, or plasma to detect three unique *M. tuberculosis* complex antigens^19^. One of these antigens is an *M. tuberculosis* cell wall complex carbohydrate antigen, while the other two are patented, proprietary targets. This study determined if the Lionex-test could detect *M. bovis* in milk^19^.

Michigan is one of the few US states with a known, sustained BTB reservoir in white-tailed deer and ongoing BTB outbreaks in cattle. Michigan has spent over $160 million in recent years to control BTB without successfully eradicating this disease from the known deer reservoir^20^. In this study, in collaboration with Michigan Department of Agriculture and Rural Development (MDARD), we tested specimens from cattle suspected to have BTB derived from depopulated Michigan cattle during a BTB outbreak from 2014–2015. Tissue, blood, milk (from dairy herd), and urine specimens were collected from suspect cows for BOVIGAM, necropsy, histology, culture, and PCR at a Michigan slaughter plant or at the Michigan State University Veterinary Diagnostic Laboratory (MSUVDL). We performed an animal level analysis using milk and urine samples to determine the efficacy of the LAM-test and Lionex-test. Our results revealed the potential of the LAM-test and Lionex- test as a POC tests for BTB.

## Results

### LAM-test

**Figure 1:**
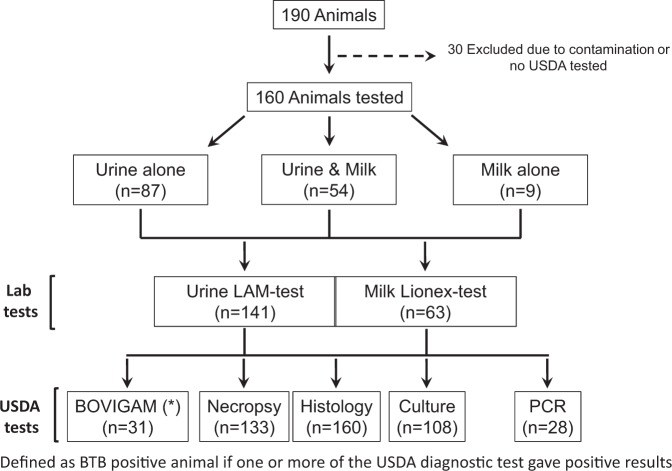
Number of animals included in this study and lab and USDA tests performed. In this study, urine and/or milk was obtained from 190 animals suspicious of having BTB. Of these, 160 animals were post-mortem tested using at least one of the USDA standard BTB test (BOVIGAM, Necropsy, Histology, culture and/or PCR). For the purpose of this study, an animal with USDA positive BOVIGAM, necropsy, histopathology, culture, and/or PCR results was considered to have positive BTB status. Urine and milk were used from these animals to test the accuracy of the LAM-test, and milk to test the accuracy of the Lionex-test when compared to the USDA standard tests performed for this study. In figure: (*) The bovine interferon gamma assay may be used in cattle herds only, and only when approved by the Chief State Animal Health Official and AVIC as an official test for use in the State and with the concurrence of the DTE and the VS Regional Tuberculosis Epidemiologist, as outlined in Part III, A.4. of ref. ^3^.

**Figure 2:**
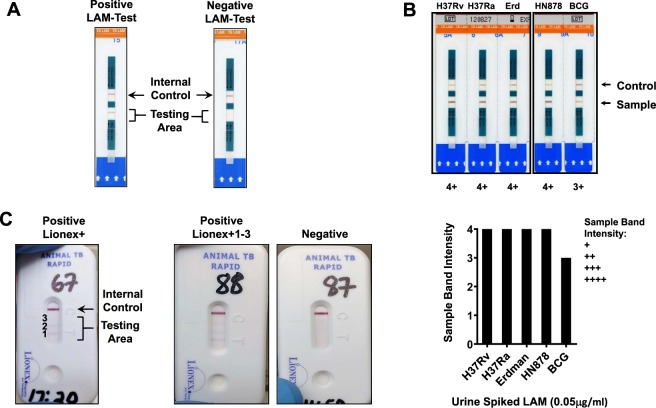
Examples of positive and negative test results for the LAM-test and Lionex-test. (**A**) The LAM-test has two zones, the control zone, which always shows a purple positive band and the testing zone, where when a purple band appears indicates a positive result for LAM detection in the analyzed specimen. (**B**) *M. tuberculosis* complex LAM (5 μg) was spiked in 1 ml urine of urine and LAM-test performed to determine if this test can detect LAM from *M. tuberculosis* complex including attenuated (H_37_R_a_), virulent (H_37_R_v_, Erdman) and hypervirulent (HN878) strains of *M. tuberculosis* and *M. bovis* BCG. A positive result is considered by band intensity values read between +1 (minimum) and +4 (maximum). (**C**) The Lionex-test has two zones, the control zone marked by a C and the testing zone marked by a T. Within the testing zone there are three *M. tuberculosis* complex antigens, each of them defined by a positive purple band if antibodies against these antigens are present in the sample being tested. One strong line appears in the Test zone together with the control line (positive result). Two strong bands appear in the test zone together with the control line (positive result). No bands present in the test zone and a band alone appears in the control line (negative result).

Samples were collected from 190 animals. Due to contamination or no paired USDA testing results, 30 animals were excluded from analysis. Urine (n = 141) and milk (n = 63) samples were collected, frozen, transported to The Ohio State University, thawed, and then tested from 160 BTB-suspected animals, with 54 animals contributing both urine and milk samples (Fig. 1). For the purpose of this study, the LAM-test was performed in urine and milk (e.g. milk plasma phase) samples (Fig. 2A). This test was first proven to detect LAM from *M. tuberculosis* complex strains in laboratory setting, including attenuated (H_37_R_a_), virulent (H_37_R_v_, Erdman), hypervirulent (HN878) *M. tuberculosis* and importantly for this study, *M. bovis* (BCG) species (Fig. 2B).

For the purpose of this study, an animal with USDA positive BOVIGAM, necropsy, histopathology, culture, and/or PCR results was considered to have positive BTB status. The proportion of positive and negative results for BTB in the animals studied was 103 (73%) and 38 (27%), respectively. Subsequently, LAM-tests were performed in 141 urine samples and results read at 25 min. Of these, 29.1% (41/141) tested positive when read at 25 min, and 80.9% (114/141) tested positive when read at 1 or 24 h; results from 1 and 24 h were identical, and were therefore interchangeable for the purpose of data analysis. All of the animals received at least two of the USDA standard tests to determine BTB positivity (BOVIGAM, necropsy, histology, culture, PCR). BOVIGAM was used in only 31 animals. Of the standard BTB tests performed by USDA in urine collected animals, 6 animals were tested by only one test, 31 animals were tested by two tests, 36 animals were tested by three tests, 68 animals were tested by four tests, and no animals received all five tests. These USDA standard tests combined were used to determine overall BTB results of animals and subsequently the sensitivity and relative specificity of the LAM-test and Lionex-test. See Supplemental Table S1 for the detailed testing performed per each animal studied.

At 25 min read, the urine LAM-test exhibited low sensitivity (30.1%), high relative specificity (73.7%) based on the BTB status. The LAM-test sensitivity, when compared to USDA diagnostic tests performed ranged from 6.3 to 40.3% (Table 1). The LAM-test relative specificity ranged from 72.2 to 84.6% (Table 1). Overall, the LAM-test had poor agreements with BTB status and USDA diagnostic tests, with kappa values of 0.025 for BTB status, and a range from −0.084 to 0.114 for individual USDA diagnostic test.

**Table 1:** Performance of the LAM-test in urine against USDA reference tests.

Type of Test	Urine LAM 25 min		Urine LAM 1 hr/24 hr	
	Negative	Positive	Negative	Positive
**BOVIGAM**				
13 Negative	11 (84.6)	2 (15.4)	0 (0)	13 (100)
16 Positive	15 (93.8)	1 (6.3)	1 (6.3)	15 (93.8)
**Necropsy**				
44 Negative	32 (72.7)	12 (27.3)	7 (15.9)	37 (84.1)
72 Positive	43 (59.7)	29 (40.3)	17 (23.6)	55 (76.4)
**Histology**				
42 Negative	31 (73.8)	11 (26.2)	7 (16.7)	35 (83.3)
98 Positive	68 (69.4)	30 (30.6)	20 (20.4)	78 (79.6)
**Culture**				
36 Negative	26 (72.2)	10 (27.8)	6 (16.7)	30 (83.3)
62 Positive	43 (69.4)	19 (30.7)	11 (17.7)	51 (82.3)
**PCR**				
0 Negative	0 (0)	0 (0)	0 (0)	0 (0)
22 Positive	15 (68.2)	7 (31.8)	5 (22.7)	17 (77.3)
**BTB Status**				
38 Negative	28 (73.7)	10 (26.3)	7 (18.4)	31 (81.6)
103 Positive	72 (69.9)	31 (30.1)	20 (19.4)	83 (80.6)

*Note: Comparison of LAM-test results (at 25 min and 1 h & 24 h) performed in urine samples with the other USDA tests results obtained from the same animals. Breakdown of true positive and negative LAM-tests, as well as false positive and false negative LAM-test results. Includes physical number of tests performed for each category along with percentage of total number in parentheses. Results suggest not only low sensitivity at 25 min due to high number of false negative LAM-tests, but also overly sensitive at 1 h or 24 h, with an increase in false positive LAM-test results when compared to the tests performed by USDA.

For the purpose of this study, an animal with USDA positive necropsy, histopathology, culture, and/or PCR results was considered to have positive BTB-status, and positive BOVIGAM as exposed/infected. An animal was considered to have a negative BTB status, if this tested negative on all USDA tests performed.

The 1 and 24 h reading reverted the trend observed at 25 min, showing high sensitivity (80.6%), low relative specificity (18.40%), yet had poor agreements with both BTB and USDA diagnostic tests. The sensitivity for each test increased to range from 77.3 to 93.8% and the relative specificity ranged from 0 to 16.7%. The kappa values were −0.01 for BTB status, with a range from −0.085 to −0.012 for individual USDA diagnostic test.

Similarly, at 25 min read, the milk LAM-test presented low sensitivity (4.4%), moderate relative specificity (87.5%), and overall had no significant agreements with BTB and USDA diagnosis tests (kappa values −0.033 to 0.118) (Table 2).

**Table 2:** Performance of the LAM-test in milk against USDA reference tests.

Type of Test	Milk LAM 25 min		Milk LAM 1 h/24 h	
	Negative	Positive	Negative	Positive
**BOVIGAM**				
1 Negative	1 (100)	0 (0)	1 (100)	0 (0)
6 Positive	6 (100)	0 (0)	6 (100)	0 (0)
**Necropsy**				
22 Negative	20 (90.9)	2 (9.1)	20 (90.9)	2 (9.1)
40 Positive	38 (95)	2 (5)	37 (92.5)	3 (7.5)
**Histology**				
21 Negative	19 (90.5)	2 (9.5)	19 (90.5)	2 (9.5)
41 Positive	39 (95.1)	2 (4.9)	38 (92.7)	3 (7.3)
**Culture**				
20 Negative	18 (90)	2 (10)	18 (90)	2 (10)
10 Positive	8 (80)	2 (20)	8 (80)	2 (20)
**PCR**				
0 Negative	0 (0)	0 (0)	0 (0)	0 (0)
26 Positive	25 (96.2)	1 (3.9)	24 (92.3)	2 (7.7)
**BTB Status**				
16 Negative	14 (87.5)	2 (12.5)	14 (87.5)	2 (12.5)
46 Positive	44 (95.7)	2 (4.4)	43 (93.5)	3 (6.5)

*Note: Comparison of LAM-test results (at 25 min and 1 h & 24 h) performed in milk samples with the other USDA tests results obtained from the same animals. Breakdown of true positive and negative LAM-tests, as well as false positive and false negative LAM-test results. Includes physical number of tests performed for each category along with percentage of total number in parentheses. Results suggest low sensitivity at both 25 min and 1 h or 24 h.

For the purpose of this study, an animal with USDA positive necropsy, histopathology, culture, and/or PCR results was considered to have positive BTB-status, and positive BOVIGAM as exposed/infected. An animal was considered to have a negative BTB status, if this tested negative on all USDA tests performed.

Milk samples read at 1 and 24 h maintained low sensitivity (6.5%), high relative specificity (87.5%) (Table 2), and no significant agreements with BTB and USDA diagnosis tests with kappa values at −0.156 to 0.118.

Overall, for urine and milk samples, reading the LAM-test at 25 min yielded low sensitivity and high relative specificity. This trend was maintained for milk samples when read at 1 h and 24 h. Alternatively, reading urine samples at 1 and 24 h significantly improved the sensitivity but lowered the relative specificity.

### Lionex-test

Lionex testing (Fig. 2C) was performed in 29 of the collected milk samples and read for Lionex+ and Lionex+1–3 results. Of these, 90.0% (26/29) tested positive for Lionex+ and 24.1% (7/29) tested positive for Lionex+1–3. In general, Lionex+ kappa values showed no agreement (−0.158 to 0.139), with overall sensitivity of 91.7% and relative specificity of 20% (Table 3). The sensitivity and relative specificity ranged between 87.5 to 100%, and 0 to 20%, respectively. For Lionex+1–3, with overall sensitivity of 16.7% and relative specificity of 40%, there was no significant agreement with BTB and USDA diagnosis tests. The sensitivity ranged from 12.5 to 50% while relative specificity ranged from 0 to 50%, respectively (Table 3).

**Table 3:** Performance of the Lionex-test in milk against USDA reference tests.

Type of Test	Milk Lionex +		Milk Lionex+1–3	
	Negative	Positive	Negative	Positive
**BOVIGAM**				
0 Negative	0 (0)	0 (0)	0 (0)	0 (0)
2 Positive	0 (0)	2 (100)	1 (50.0)	1 (50.0)
**Necropsy**				
5 Negative	1 (20.0)	4 (80.0)	2 (40.0)	3 (60.0)
24 Positive	2 (8.3)	22 (91.7)	20 (83.3)	4 (16.7)
**Histology**				
6 Negative	1 (16.7)	5 (83.3)	3 (50.0)	3 (50.0)
23 Positive	2 (8.7)	21 (91.3)	19 (82.6)	4 (17.4)
**Culture**				
3 Negative	0 (0)	3 (100)	1 (33.3)	2 (67.7)
8 Positive	1 (12.5)	7 (87.5)	7 (87.5)	1 (12.5)
**PCR**				
0 Negative	0 (0)	0 (0)	0 (0)	0 (0)
11 Positive	1 (9.1)	10 (90.9)	8 (72.3)	3 (27.3)
**BTB Status**				
5 Negative	1 (20)	4 (80)	2 (40)	3 (60)
24 Positive	2 (8.3)	22 (91.7)	20 (83.3)	4 (16.7)

*Note: Comparison of Lionex-test results performed in milk samples with the other USDA tests results obtained from the same animals. Tests were categorized into two categories, Lionex+ and Lionex+1–3. Lionex+ indicates at least one antibody was present in the milk sample, eliciting at least one visible antigen line on the test. Lionex+1–3 indicates that all three specific antibodies were present in the milk sample, eliciting three visible antigens on the test card. Neither Lionex+ or Lione+1–3 analysis elicited statistically significant results. However, Lionex+ true positive trends suggest future studies of larger sample sizes may further validate this assay.

For the purpose of this study, an animal with USDA positive necropsy, histopathology, culture, and/or PCR results was considered to have positive BTB-status, and positive BOVIGAM as exposed/infected. An animal was considered to have a negative BTB status, if this tested negative on all USDA tests performed.

## Discussion

Currently there is a lack of accurate, field deployable standard field tests for veterinarians to establish quickly in the field if an animal is truly infected with *M. bovis* and/or has active BTB. Additional methods include post-mortem testing (necropsy, histology), methods that are challenging to perform and/or require time and expertise, or are costly (*i.e*. BOVIGAM, culture, PCR). In an attempt to address if two point of care tests could be useful to diagnose BTB, we evaluated the efficacy of the Alere Determine LAM TB Urine Antigen Test (LAM-test, initially designed for human TB) and Lionex Animal TB Rapid Blood Test (Lionex-test) in accurately diagnosing BTB in cattle using urine and milk samples, two non-invasive and easily obtainable samples. The goal was to validate these rapid, field-based, easy-to-use, and relatively low cost (~$3.5/test USD) diagnostic tests for BTB surveillance in the US and their potential suitability in high BTB endemic countries. Better POC testing in the field for BTB could fulfill significant agricultural and public health needs, enable rapid detection, and aid in preventing transmission of BTB between animals and from animals to humans. However, our comparisons between the LAM-test (using urine or milk) and USDA standard diagnostic tests yielded poor agreement. The same milk samples did not elicit significant agreement with the Lionex-test (n = 29), although positive trends were present. Though kappa values were low for Lionex+ readings, high sensitivities for necropsy, histology, culture, and PCR (91.7%, 91.3%, 87.5%, 90.9%, respectively) suggest the validity of this test needs to be further studied. These trends dissipate when the test was read for Lionex+1–3 results (Table 3). Due to the overall poor agreement from the results (kappa value <0.2), increasing the sample size may not likely demonstrate the utility of the LAM-test and Lionex-test. Further studies will need to address modifications on these two diagnostic test methods to improve their sensitivity and relative specificity (>80%). These may include performing the test in the field as true POC (where urine/milk is collected *vs*. using frozen samples) and comparing to post-mortem evaluation and gold standard culture and PCR, study location (BTB endemic *vs*. non-endemic area), sample transport and preparation, and evaluate test’s performance dependency on the nature of *M. tuberculosis* complex strains, among others.

In our study, we first validated in the lab setting that the LAM-test can detect LAM from different *M. tuberculosis* complex species, including *M. bovis*. In this regard, it is not exactly described the nature of the LAM epitopes(s) that this test recognizes, but it is reported that detects mannose-capped LAM (or ManLAM) from *M. tuberculosis* complex (which includes *M. tuberculosis* and *M. bovis* among other species) at 97% relative specificity^16,17^. Urine spiked with in-house purified *M. bovis* BCG LAM was detected by the LAM-test at similar levels (+3 band intensity) as we observed for purified LAMs from several *M. tuberculosis* laboratory strains (attenuated H_37_R_a_, virulent H_37_R_v_, virulent Erdman, and hypervirulent HN878), which gave a band intensity of +4. Thus, this test should be suitable to detect *M. bovis* LAM in urine.

Because the LAM-test was originally designed for human use, test results were read at the manufacturer-recommended 25 min, but also at 1 and 24 h to determine if efficacy varied as time-after-exposure progressed. Results at the 25 min differed from results at 1 and 24 h, however, 1 and 24 h results showed no difference. Our LAM-test results, for urine and milk samples, produced inconsistent results at the various time intervals studied. The high negative-to-positive conversion rate between time points may suggest that the LAM-test is reactive to additional unidentified microbial antigens found in the cattle’s urine and/or there is an increase of unspecific binding overtime. Based on these results, the LAM-test does not appear to be an effective test for BTB using urine. Interestingly, by extending the reading time to 1 h, increased positive results were obtained. Thus, the LAM-test could be potentially considered as a screening test, which positiveness will need to be confirmed by a more specific test.

The original Lionex-test was developed for the detection of human TB^19^; however, the one used in this study was specifically designed for BTB. The Lionex-test detects antibodies present in blood, serum, and plasma against three different antigens of the *M. tuberculosis* complex cell wall. These antigens are ManLAM, a mixture of recombinant cell wall proteins (non-specified by the company), and a third non-disclosed proprietary antigen. Since the majority of antigens in the Lionex-test are of proprietary knowledge, we relied on the company quality controls of this test, which is commercially available and sold for the detection of BTB in animals^19^. Thus, a Lionex-test positive result is defined as any detection of antibodies against any of the three antigens present in this test. Given this fact, there are a total of 7 different possible positive results for this test, with there being 3 single positive results (1; 2; or 3) and 4 combination positive results (1 and 2; 1 and 3; 2 and 3; and 1, 2, and 3). Despite the number of possibilities, there were only 2 combinations that were observed on the samples studied. Lionex+ result was defined when a milk sample on the Lionex-test gave positive at least for one of the three antigens. However, positive results for antigen 1 and 2 always displayed together. Lionex+1–3 result was defined when a milk sample on the Lionex-test gave positive for all three antigens at the same time. No other combinations were present from all samples tested. These specific combinations might suggest an interrelationship between the different antigens. This finding could be specific to *M. bovis* or other members of the *M. tuberculosis* complex. Moreover, *Mycobacterium avium* subspecies *paratuberculosis* or Johnes disease is of particular concern given that the 2007 Dairy HAHMS study found approximately at least one cow positive for Johnes in 68% of US dairy herds^21^. It is unknown if the Lionex-test could be cross-reacting with these bacteria and may warrant further investigation to clarify the relationship.

These study presented several limitations. It could have benefited from being able to have all comparative tests performed on each animal to increase the accuracy of the diagnosis. Unfortunately, there were a variety of challenges with sample collection which impacted the number of milk samples available for the LAM-test and Lionex-test testing. This study also would have benefited from a larger negative control sample size, i.e. healthy, certified BTB free cattle from the same region of the cattle suspected of BTB to evaluate the true specificity of the tests. Additional comparisons were made between tests with theoretical negative control samples and the levels of agreement dramatically improved, especially for the Lionex-test. These results, however, were not included in this study due the concerns of artificially increasing the pool of negative BTB samples to benefit the statistics. A concern is the issue with BTB serology sensitivity. The Lionex-test is a serological test and its sensitivity for BTB could greatly vary depending on the circumstances (e.g. number of infected animals, previous CFT/CCT tests, BCG vaccination, duration and extent of the infection, etc.)^22,23^. For example, the interval of time between a stimulus (e.g. infection, CFT test-PPD administration) and the sampling to perform the Lionex-test could be important because the anamnestic response is better between 8 days and 28 days^24–27^. As the Lionex-test was mainly designed for blood, serum and plasma samples (from cattle with active BTB disease), in future studies will be also necessary to compare sensitivity results of the Lionex-test on milk with the Lionex-test on blood compared to the standard USDA diagnostic tests.

Another limitation is that the LAM-test and the Lionex-test were performed using frozen samples and thus, it may have an impact on the performance of these tests. Freeze/thaw may alter the detection of LAM in urine/milk by the LAM-test, and may also alter the functionality of antibodies detected by the Lionex-test in milk.

This study was carried out in a highly infected population where the positive predicted values (PPV) of the necropsy and histological USDA tests were accurate. However, caution must be taken defining the status based only on these tests in a low prevalence population^3^. The BOVIGAM test sometimes also shows a high false positive rate for potential exposure/infection (e.g. due to herds exposed to environmental non-tuberculous mycobacteria)^3,4^. Thus, combined necropsy, histology and BOVIGAM PPVs can become poor in low prevalence populations and thus, PCR or culture should be used on these populations to confirm the BTB status of the animal. Indeed, in our study we did not have any animal that just received the BOVIGAM test alone, and the ones that received this test, their result was confirmed by other USDA diagnostic tests (Table 1, see as examples, animals 120–126). Conversely, there were 7 animals (Table 1, animals 2, 5, 8, 11, 15, 17, and 36) that gave BOVIGAM negative, but tested positive by other USDA diagnostic tests. In this instance, these animals were diagnosed as BTB positive based on their culture positive result. In this regard, animals in this study were already selected for slaughtering as suspicious of having BTB, thus the majority of diagnostic tests performed by USDA in these animals were post-mortem.

Hence, additional studies in BTB endemic areas are needed to provide more thorough data about the use of the LAM-test (in urine and milk) and Lionex-test (in milk) in the field. Furthermore, future studies will need to compare the effectiveness of the Lionex-test to currently used CFT screening test and CCT confirmatory test in dairy cattle, as well as BOVIGAM. This is also true for the LAM-test. If either test is to become the new means of testing for BTB in cattle, then it must be compared to USDA and European Union’s current standards; otherwise, it will fail to gain traction. This study was unable to conduct these comparisons because the samples for testing were collected during herd depopulations.

Under our experimental conditions and following manufacturer’s guidelines, the LAM-test is not considered valid for accurate diagnosis of BTB in cattle using milk or urine samples. The use of the LAM-test for BTB diagnosis in urine and milk samples will need further evaluation, as a recent report indicate that enzymatic treatments of LAM-spiked urine and milk could increase the efficiency of the LAM-test in detecting LAM in these samples^28^. This is an active research in our laboratories.

Though the Lionex-test did not produce statistically significant results, the trends observed may suggest the Lionex-test in milk was in closer agreement to the USDA standard diagnostic testing performed. The Lionex-test, however, showed some trends suggesting that the easily obtainable milk samples may be a viable option for non-invasive, rapid diagnosis of *Mycobacterium bovis* infection in cattle. Further studies will need to be performed to verify that the Lionex-test is up to this challenge.

## Methods

### Animals

The study was conducted using animals recruited from depopulated, BTB-suspected cattle in northeastern Michigan which experienced a BTB outbreak in 2015. This BTB area formed by Alcona, Alpena, Montmorency, Oscoda and Presque Isle County is classified as a Modified Accredited Zone (a State or zone of a State that must have had a TB prevalence less than 0.1% of the total number of cattle and bison herds for the most recent year) by the USDA Bovine Tuberculosis Eradication Uniform Methods and Rules^3^. Samples were collected from 190 animals. Due to contamination or no USDA diagnostic testing, 30 animals were excluded from analysis. Urine (n = 141) and milk (n = 63) samples were collected, frozen, transported to The Ohio State University, thawed, and then tested from 160 BTB-suspected animals, with 54 animals contributing both urine and milk samples (Supplemental Table S1). BTB infection was determined using lung tissue culture, histology, necropsy, BOVIGAM, and PCR methods at MSUVDL. Negative control milk samples came from a local Ohio dairy that was certified BTB free. To obtain negative control urine samples, urine was obtained from the bladder from BTB free cattle. All samples were filter-sterilized using low protein binding 0.2 μm filter-membranes, aliquoted and frozen at −80 °C until processing.

### Alere determine LAM TB Urine or milk Antigen Test (LAM-test)

The LAM-test (Alere Determine, Alere, Waltham, MA) is an immunochromatographic test for the qualitative detection of LAM antigen of mycobacteria in urine^29,30^. The LAM-test employs highly purified antibodies specific to the major lipoglycan antigen of the Genus *Mycobacterium*, LAM. Although this test was originally designed for human TB detection using urine^16,31^, here we evaluated this test for the diagnosis of BTB in cattle using urine and milk. To analyze milk samples, 1 ml of milk was centrifuged for 10 min at 5,000 × *g* to obtain the milk plasma phase (supernatant) for the testing.

Prior to testing, specimens were removed from −80 °C storage, and thawed on ice. Following the manufacturer instructions, 60 μl of urine or milk (plasma phase) was added into the sample pad on the test strip and kept at room temperature for 25 min before the first reading. For the purpose of this study, band intensity was not taken into consideration when determining positive results; if a band appeared, the test strip was considered positive (Fig. 1A). Test strips were read again at 1 and 24 h to determine if additional incubation time positively or negatively altered the performance of this test. All test results were interpreted according to the commercial LAM-test test manual.

To show that the LAM-test can detect *M. bovis* LAM, 1 ml of urine from healthy animals was spiked with 0.05 μg of purified LAM from different *M. tuberculosis* complex strains [attenuated H_37_R_a_, virulent H_37_R_v_ and Erdman, hypervirulent HN878 *M. tuberculosis* strains and *M. bovis* BCG (BCG) strain] obtained as we described^32–34^. Strains within the *M. tuberculosis* complex have a unique mannose-capped LAM (or ManLAM), where *M. tuberculosis*, *M. bovis* and *M. bovis* BCG ManLAMs have similar (no identical) structures^35^.

### The lionex bovine TB-ST Rapid Test (Lionex-Test)

The Lionex-test is a lateral flow immuno-chromatogra-phic, membrane-based screening test for the rapid detection of antibodies (IgG/IgM/IgA) in whole blood, plasma, or serum of animals (Lionex diagnostic and therapeutic GmbH Company, Germany)^19^. This test contains a membrane coated with: i) a special antibody binding protein, conjugated to colloidal gold particles (conjugate); ii) three test lines, two lines consisting of two specific recombinant antigens from *M. bovis*, and the third containing a highly purified mycobacterial cell wall antigen (LAM); and iii) a control line consisting of an antibody binding protein, indicating that the test has been properly performed. The sensitivity and relative specificity of the Lionex-test was measured by the manufacturer using specific antigens from *M. bovis* in blood from cattle. The manufacturer-stated specificity of the Lionex-test was more than 95% and the sensitivity 74.29%^19^.

To test the milk, 20 µl of milk plasma phase, obtained by centrifugation as above indicated, was added to the Lionex-test well making sure to avoid the milk fat; and immediately followed by 2 drops of the diluent buffer provided. After 5 min, an additional drop of the diluent was added to the sample well, followed by a 20 min incubation at room temperature.

Following the manufacturer instructions, a Lionex-test positive result is when a band appears within the testing area (T) next to the ‘1’ [detecting antibodies against an *M. tuberculosis* cell wall complex carbohydrate antigen, LAM), ‘2’ or ‘3’ (detecting antibodies against a mixture of recombinant antigens) indicators alone, or when multiple bands appear in combination (Fig. 1B). Negative results are when bands do not appear next to ‘1, 2 or 3’ indicators. The ‘C’ band corresponds to the test’s internal positive control and always need to appear to validate the test (Fig. 1B). Given the numerous positive combinations from the Lionex-test, multiple definitions were created to interpret the results. A negative result was defined as a milk sample that did not elicit appearance of any of the three antigen indicators (‘1, 2, or 3’) contained on the Lionex-test. Lionex+ was defined as a milk sample that tested positive for at least one of the three antigen indicators. However, indicator ‘1’ and ‘2’ always appeared together and became the modified definition for Lionex+. Lionex+1–3 was defined as a milk sample that tested positive for all three antigen indicators. Additional combinations were explored but failed to yield results.

### Tissue necropsy, histology, culture, BOVIGAM, PCR

All these USDA diagnostic tests were performed by the USDA National Veterinary Services Laboratories following their standard test procedures. Necropsy was performed by visual examination of tissues to determine BTB by a USDA accredited veterinarian. Tissue samples were collected from BTB-suspected and subsequently ground, digested, and concentrated for histologic examination. After the animal was sacrificed, tissue samples were cut to 2 mm thick subsample and immediately fixed in formalin to preserve cellular detail. One-part tissue to 10 parts formalin was used to adequately fix tissue^36,37^. Formalin-fixed tissues were processed and stained with hematoxylin and eosin. Any granulomatous lesions were then stained with a modified acid fast procedure and an auramine orange/acridine orange procedure^36,37^. Positive fluorescence required further examination via Zeihl-Neelsen staining methods^38^.

Fresh tissues for culture of mycobacteria were first screened for visible lesions. Tissues were decontaminated with a sodium hydroxide solution to remove fungal and bacterial contaminants. Samples were then processed and cultured using the mycobacterial growth indicator tube (MGIT) system (Becton Dickinson, Franklin Lakes, NJ). Media was prepared according to manufacturer’s guidelines, with the addition of erythromycin 6.0 μg/mL. Subsequent inoculations, acid-fast stains, and observations also followed manufacturer’s guidelines^39^.

A commercial BOVIGAM TB Kit (Applied Biosystems, Foster City, CA), was used to test blood samples in this study. Briefly, blood samples were incubated overnight with antigens, such as tuberculin purified protein derivative (PPD), to stimulate lymphocytes to produce IFN-γ. IFN- γ in the plasma supernatants of each blood aliquot was then further determined using a sandwich ELISA. IFN- γ present in the sample bound to anti-bovine IFN- γ monoclonal antibodies and was further visualized with a secondary anti-IFN- γ antibody labeled with an enzyme that generated a color signal. Color development was proportional to the amount of bound IFN- γ^40^.

Acid-fast bacilli stain and PCR were performed on tissue specimens in addition to histopathology and PCR^41,42^. PCR tests were conducted according to the original procedure developed for *M. bovis* identification, with incorporation of subsequent modifications. Briefly, a crude tissue extract was prepared from two paraffin sections (5 μm each) that were placed in a 1.5 ml microcentrifuge tube and pelleted by centrifugation at 16,000 × *g* for 1 min, followed by the addition of 200 μl water containing 0.5% Tween 20. The tube was then subjected to two cycles of a 10 min boil followed by snap freezing (ethanol on dry ice). After a third 10 min boil the sample was centrifuged at 3000 × *g* for 20 min and 10 μl of the supernatant was used for each PCR test.

For the purpose of this study, an animal with USDA positive BOVIGAM, necropsy, histopathology, culture, and/or PCR results was considered to have positive BTB status. An animal was considered BTB negative when it did not test positive on any of these five USDA diagnostic tests reported. These combined USDA diagnostic tests were used to determine overall BTB results of animals and subsequently the sensitivity and relative specificity of the LAM-test and Lionex-test. Sensitivity analysis (true positive rate) measured the proportion of correctly identified positives by any of the five USDA diagnostic test reported. As all the samples originated from a BTB suspected population, specificity as such could not be estimated because the studied population could not be guaranteed of being truly BTB free despite negative reference tests. Thus, the proportion of samples that tested negative by any of the five USDA diagnostic test reported were used to define the relative specificity.

### Data analysis

Data analyses were conducted using SAS (9.4, SAS Inst. Inc., NC, US). Test sensitivity (proportion of positive result found among infected animals) and relative specificity (proportion of negative result among non- infected animals) of the LAM-test on urine and milk, and the Lionex-test on milk, were estimated based on the BTB status and five individual USDA diagnostic tests. The agreement among different diagnostic tests was evaluated using Cohen’s Kappa coefficient.

### Ethics approval and consent to participate

Under The Ohio State University Institutional Animal Care and Use Committee (IACUC) waiver and permission, all samples were obtained post-mortem during a depopulation of cattle suspected to be infected by *M. bovis* performed by the Michigan Department of Agriculture and Rural Development, and the Diagnostic Center for Population and Animal Health at Michigan State University under USDA supervision.

## Supplementary information

Supplementary Table S1.

## Data Availability

All data generated and/or analyzed during the current study are available in this published article.
