# Supplementary material for: Accuracy of Two Point-of-Care Tests for Rapid Diagnosis of Bovine Tuberculosis at Animal Level using Non-Invasive Specimens
Source: Sci Rep. 2020 Mar 25;10:5441. doi: 10.1038/s41598-020-62314-2 (PMC7096388; doi:10.1038/s41598-020-62314-2)
Supplement: Supplementary file 1 — Supplementary Table S1. [file 41598_2020_62314_MOESM1_ESM.pdf]

Supplemental Material

Accuracy of Two Point-of-Care Tests for Rapid Diagnosis of Bovine Tuberculosis at Animal Level using Non-Invasive Specimens

Holden V Kelley, Sarah M Waibel, Sabeen Sidiki, Cristina Tomatis-Souverbielle, Julia M Scordo, W. Garret Hunt, N. Barr, R. Smith, Sayeed N Silwani, James J Averill, Susan Baer, Janet Hengesbach, Vedat O Yildiz, Xueliang Pan, Wondwossen A Gebreyes, Joan-Miquel Balada-Llasat, Shu-Hua Wang, and Jordi B. Torrelles

Supplemental Table S1. Summary Table of Procedures Performed on Depopulated Animals. Table showing the summary of the results for all the procedures performed in the animals involved in this study.

Additional Information: Supplemental Table S1*

Summary Table of Procedures Performed on Depopulated Animals

Animal ID	Sample Type	LAM+ 25min	LAM+ 1h/24h	Lionex+	Lionex 1-3	B	N	H	C	P	BTB Status
1-U-MI	U	-	+			-	-	-	-		-
2-U-MI	U	-	+			-	+	+	+		+
3-U-MI	U	-	+			+	+	+	+		+
4-U-MI	U	-	+			+	+	+	+		+
5-U-MI	U	-	+			-	+	+	+		+
6-U-MI	U	-	+			+	+	+	+		+
8-U-MI	U	-	+			-	-	-	+		+
9-U-MI	U	-	+			+	+	+	+		+
10-U-MI	U	-	+			+	+	+	+		+
11-U-MI	U	-	+			-	+	+	+		+
12-U-MI	U	-	-			+	+	+	+		+
13-U-MI	U	-	+			-	+	+	+		+
14-U-MI	U	-	+			+	+	+	+		+
15-U-MI	U	-	+			-	+	+	+		+
16-U-MI	U	-	+			+	+	+	+		+
17-U-MI	U	-	+			-	+	+	+		+
18-U-MI	U	-	+					+	+		+
19-U-MI	U	-	+					+	+		+
20-U-MI	U	-	-					+	+		+
21-U-MI	U	-	+					+			+
22-U-MI	U	-	-					+	+		+
23-U-MI	U	-	+					+	+		+
24-U-MI	U	-	+					+	+		+
25-U-MI	U	-	+					+	+		+
26-U-MI	U	-	+					+			+
27-U-MI	U	-	+					+			+
28-U-MI	U	-	+					+	+		+
30-U-MI	U	-	+			+	+	+	+		+
34-U-MI	U	-	+			+		+	+		+
35-U-MI	U	-	+			+		+	+		+
36-U-MI	U	-	+			-		+	+		+
58-U-MI	U	-	-				-	-	-		-
59-U-MI	U	-	-				+	+	+	+	+
60-U-MI	U	-	-				+	+	+		+
60-M-MI	M	-	-	-	-		+	+	+		+
61-U-MI	U	-	-				+	+	-		+
62-U-MI	U	-	-				-	-	-		-
63-U-MI	U	-	+				-	-	-		-
63-M-MI	M	-	-	+	+		-	-	-		-
64-U-MI	U	+	+				+	+	+	+	+
64-M-MI	M	+	+	+	-		+	+	+	+	+
65-U-MI	U	+	+				+	+	+		+
66-U-MI	U	-	-				-	-	-		-

Animal ID	Sample Type	LAM+ 25min	LAM+ 1h/24h	Lionex+	Lionex 1-3	B	N	H	C	P	BTB Status
66-M-MI	M	-	-	+	+		-	-	-		-
67-M-MI	M	-	-	+	-		+	+	+		+
68-U-MI	U	-	-				+	+	+		+
68-M-MI	M	-	-	+	-		+	+	+		+
69-U-MI	U	-	-				+	+	+	+	+
69-M-MI	M	-	-	+	-		+	+	+	+	+
70-M-MI	M	-	-	+	-		+	+	+		+
71-U-MI	U	-	-				+	+	+		+
71-M-MI	M	+	+	+	-		+	+	+		+
72-U-MI	U	-	-				-	-	-		-
74-U-MI	U	-	-				+	+			+
75-U-MI	U	-	-				+	+			+
75-M-MI	M	-	-	+	-		+	+			+
76-M-MI	M	-	-	+	-		+	+			+
77-U-MI	U	-	-				-	-			-
77-M-MI	M			+	+		-	-			-
78-M-MI	M	-	-	-	-		-	-			-
79-U-MI	U	-	-				+	+			+
79-M-MI	M	-	-	+	+		+	+			+
80-U-MI	U	-	-				+	+			+
80-M-MI	M	-	-	+	-		+	+			+
81-U-MI	U	+	+				+	+			+
81-M-MI	M	-	-	+	-		+	+			+
82-U-MI	U	+	+				+	+			+
82-M-MI	M	-	-	+	-		+	+			+
83-U-MI	U	-	-				+	+			+
83-M-MI	M	-	-	+	-		+	+			+
84-U-MI	U	+	+				-	-			-
85-M-MI	M	-	-	+	-		+	+		+	+
86-U-MI	U	-	-				+	+		+	+
86-M-MI	M	-	-	+	-		+	+		+	+
87-U-MI	U	+	+				+	+		+	+
87-M-MI	M	-	-	-	-		+	+		+	+
88-U-MI	U	+	+				+	+		+	+
88-M-MI	M	-	-	+	+		+	+		+	+
89-U-MI	U	+	+				+	+		+	+
89-M-MI	M	-	-	+	-		+	+		+	+
91-U-MI	U	-	-				+	+		+	+
91-M-MI	M	-	-	+	+		+	+		+	+
92-U-MI	U	-	-				+	+		+	+
92-M-MI	M	-	-	+	-		+	+		+	+
93-U-MI	U	+	+				+	+		+	+
93-M-MI	M	-	-	+	-		+	+		+	+

Animal ID	Sample Type	LAM+ 25min	LAM+ 1h/24h	Lionex+	Lionex 1-3	B	N	H	C	P	BTB Status
94-U-MI	U	-	+				+	+			+
95-U-MI	U	+	+				+	+			+
96-U-MI	U	+	+				+	+			+
98-U-MI	U	+	+				+	+			+
100-U-MI	U	-	-				-	-			-
102-M-MI	M	-	-	+	+	+	+	+	+	+	+
103-U-MI	U	+	+			-	-	-	-		-
104-U-MI	U	+	+			-	-	-	-		-
105-U-MI	U	-	+			+	+	-			+
105-M-MI	M	-	-	+	-	+	+	-			+
106-U-MI	U	-	+				-	-	-		-
107-U-MI	U	-	+				-	-	-		-
108-U-MI	U	-	+				-	-	-		-
109-U-MI	U	+	+				-	-	-		-
110-U-MI	U	-	+				-	-	-		-
111-U-MI	U	-	+				-	-	-		-
112-M-MI	M	+	+	+	-		-	-	-		-
113-U-MI	U	-	+				-	-	-		-
114-U-MI	U	+	+				-	-	-		-
115-U-MI	U	+	+				-	-	-		-
116-U-MI	U	+	+				-	-	-		-
117-U-MI	U	-	+				-	-	-		-
118-U-MI	U	+	+				-	-	-		-
119-U-MI	U	-	+				-	-	-		-
119-M-MI	M	-	-				-	-	-		-
120-U-MI	U	-	+			-	-	-	-		-
121-U-MI	U	-	+			+	+	+		+	+
121-M-MI	M	-	-			+	+	+		+	+
122-U-MI	U	-	+			+	+	+		+	+
123-U-MI	U	+	+			+	+	+		+	+
123-M-MI	M	-	-			+	+	+		+	+
124-U-MI	U	-	+			+	+	+	-		+
124-M-MI	M	-	-			+	+	+	-		+
125-U-MI	U	-	+			-	-	-	-		-
125-M-MI	M	-	-			-	-	-	-		-
126-M-MI	M	-	-			+	+	+		+	+
127-U-MI	U	+	+				+	+		+	+
127-M-MI	M	-	-				+	+		+	+
128-M-MI	M	-	-				+	+	-		+
129-M-MI	M	-	-				-	-	+		+
130-M-MI	M	-	-				-	-	-		-
131-U-MI	U	-	+				+	+		+	+
131-M-MI	M	-	-				+	+		+	+

Animal ID	Sample Type	LAM+ 25min	LAM+ 1h/24h	Lionex+	Lionex 1-3	B	N	H	C	P	BTB Status
132-M-MI	M	-	-				+	+		+	+
134-U-MI	U	-	+				-	-	-		+
134-M-MI	M	-	-				-	-	-		+
135-M-MI	M	-	-				+	+		+	+
136-M-MI	M	-	-				-	-	-		+
137-U-MI	U	-	+				+	+		+	+
137-M-MI	M	-	-				+	+		+	+
138-M-MI	M	-	+				+	+		+	+
139-U-MI	U	-	+				-	-	-		-
139-M-MI	M	-	-				-	-	-		-
140-U-MI	U	-	+				-	-		+	+
140-M-MI	M	-	-				-	-		+	+
141-U-MI	U	-	+				+	+		+	+
141-M-MI	M	-	-				+	+		+	+
142-U-MI	U	-	+				-	+	+		+
142-M-MI	M	-	-				-	+	+		+
143-U-MI	U	-	-				-	-	-		-
143-M-MI	M	-	-				-	-	-		-
144-U-MI	U	-	+				+	+		+	+
144-M-MI	M	-	-				+	+		+	+
145-U-MI	U	-	+				+	+		+	+
145-M-MI	M	-	-				+	+		+	+
146-U-MI	U	-	+				-	-	-		-
146-M-MI	M	-	-				-	-	-		-
147-U-MI	U	-	+				-	-	-		-
147-M-MI	M	+	+				-	-	-		-
148-M-MI	M	-	-				-	-	-		-
149-U-MI	U	-	+				-	-	-		-
149-M-MI	M	-	-				-	-	-		-
150-U-MI	U	+	+				-	+	-		+
150-M-MI	M	-	-				-	+	-		+
151-U-MI	U	-	+				-	-	-		-
151-M-MI	M	-	-				-	-	-		-
152-M-MI	M	-	-				-	-	-		-
153-U-MI	U	-	+				-	-	-		-
153-M-MI	M	-	-				-	-	-		-
154-U-MI	U	-	+				+	+		+	+
154-M-MI	M	-	-				+	+		+	+
155-U-MI	U	-	+				+	+		+	+
155-M-MI	M	-	-				+	+		+	+
156-U-MI	U	-	+				-	-			-
157-U-MI	U	-	+				-	-			-
158-U-MI	U	-	+				-	-			-

Animal ID	Sample Type	LAM+ 25min	LAM+ 1h/24h	Lionex+	Lionex 1-3	B	N	H	C	P	BTB Status
159-U-MI	U	-	+					+	+		+
160-U-MI	U	-	+					+	+		+
161-U-MI	U	-	-					+	+		+
162-U-MI	U	-	+					+			+
163-U-MI	U	-	+					+	+		+
164-U-MI	U	-	+					+	+		+
165-U-MI	U	-	+					+	+		+
166-U-MI	U	-	+					+	+		+
167-U-MI	U	-	+					+			+
168-U-MI	U	-	+					+			+
169-U-MI	U	-	+					+	+		+
170-U-MI	U	+	+				+	+	+		+
171-U-MI	U	+	+				+	+	+		+
172-U-MI	U	+	+				+	+	+		+
173-U-MI	U	+	+				+	+	+		+
174-U-MI	U	-	-				+	+	+		+
175-U-MI	U	+	+				-	-	+		+
176-U-MI	U	+	+				+	+	+		+
177-U-MI	U	+	+				+	+	+		+
178-U-MI	U	+	+				-	-	-		-
179-U-MI	U	+	+				+	+	+		+
180-U-MI	U	-	-				+	+	+		+
181-U-MI	U	+	+				+	+	+		+
182-U-MI	U	+	+				+	+	+		+
183-U-MI	U	+	+				+	+	+		+
184-U-MI	U	+	+				+	+	+		+
185-U-MI	U	+	+				+	+	+		+
186-U-MI	U	+	+				+	+	+		+
187-U-MI	U	+	+				+	+	+		+
188-U-MI	U	+	+				+	+	+		+
189-U-MI	U	+	+				+	+	+		+
190-U-MI	U	+	+				-	-	-		-

*Note: Overview of each sample used for this study. Each sample received a unique ID number, along with “urine” (U) or “milk” (M) identification. The table presents results of the LAM 25 min/1h/24h test (if applicable), results of the Lionex+/1-3 test (if applicable), and results of the following USDA standard BTB-diagnostic tests: BOVIGAM (B), necropsy (N), histology (H), culture (C), PCR (P). Based on the results of the USDA tests, the BTB-status of each sample was determined.
